# Chronic Bronchitis and Its Treatment

**Published:** 1890-03

**Authors:** 


*Chronic Bronchitis and its Treatment.* By WILLIAM MURRELL, M.D., F.R.C.P. London: H. K. LEWIS. 1889.

To the practitioner, oft perplexed in the treatment of this obstinate complaint, this small volume will prove a boon. Here are full and pleasant records of the value of a large variety of therapeutic methods, including those by medicine, spray, and inhalation, and all from the point of view of an extensive clinical experience.

